# CD20×CD3 bispecific antibody achieved significant efficacy in patients with large B-cell lymphoma relapsing after or refractory to CAR-T therapy: a systematic review and meta-analysis

**DOI:** 10.3389/fonc.2025.1641769

**Published:** 2025-08-22

**Authors:** Jing Shen, Jingyi Zhang, Zhengyu Zhu, Haobo Ma, Xiayan Li, Junpeng Zhang, Fan Zhou, Hua Tian, Jinghua Liu

**Affiliations:** ^1^ Department of Hematology, Capital Medical University Affiliated Beijing Friendship Hospital, Beijing, China; ^2^ Department of Pharmacology, Shenyang Pharmaceutical University, Shenyang, China; ^3^ Academy of Electronic Science and Technology, National University of Defense Technology, Changsha, China; ^4^ Department of Pharmacy/Evidence-Based Pharmacy Center, West China School of Medicine, Sichuan University, Chengdu, China; ^5^ Department of Endocrinology, The 961st Hospital of the Joint Logistics Support Force of the People's Liberation Army, Qiqihar, China; ^6^ Department of Hematology, General Hospital of the Northern Theater Command, Shenyang, China

**Keywords:** bispecific antibody, meta-analysis, CAR-T cell therapy, relapsed or refractory, large B-cell lymphoma

## Abstract

**Objective:**

Chimeric antigen receptor T-cell immunotherapy (CAR-T) is a preferred treatment for relapsed or refractory (R/R) large B-cell lymphoma (LBCL). Several trials have evaluated CD20×CD3 bispecific antibodies (BsAbs) as subsequent therapy in R/R LBCL. This study aimed to investigate the efficacy of CD20×CD3 BsAbs (mosunetuzumab, glofitamab, odronextamab, and epcoritamab) in patients with LBCL who experienced relapse or refractory disease following CAR-T therapy.

**Methods:**

Nine trials involving 350 participants were included, assessing the overall response rate (ORR), complete response (CR), duration of response (DOR), duration of complete response (DoCR), progression-free survival (PFS), and overall survival (OS).

**Results:**

The specific response rates for different bispecific antibody (BsAb) monotherapies were as follows: Mosunetuzumab: overall response rate (ORR) 40% and complete response (CR) 23%; Glofitamab: ORR 50-76.1% and CR 37-45.7%; Epcoritamab: ORR 54.1% and CR 36%; Odronextamab: ORR 48.3% and CR 31.7%. Upon pooled analysis, the overall ORR was 54.5% (95% CI: 43.1-65.7%) with significant heterogeneity (*P*=0.013, I²=68.24%), and the CR was 35.6% (95% CI: 29.1-42.2%) with low heterogeneity (*P*=0.33, I²=13.5%). The specific response rates for different BsAb combinations were as follows: Mosunetuzumab + Pola: ORR 57% and CR 40%; Glofitamab + Pola: ORR 77.8% and CR 44.4%; Epcoritamab + Gemox: ORR 76% and CR 45%; Glofitamab + Gemox: CR 53.8%. Upon pooled analysis, the overall ORR was 70.0% (95% CI: 56.4-82.2%) with no heterogeneity, and the CR was 44.2% (95% CI: 34.5-54.1%) with no heterogeneity. The median duration of follow-up ranged from 13 to 42 months. Data from five trials were available for duration of response (DOR) analysis: 9.7 months, 14.8 months, 19.7 months, not reached, and 2-year rate of 25%, respectively; three trials were available for duration of complete response (DoCR) analysis: one trial reported 22 months, and the others were not reached; six trials were available for median progression-free survival (mPFS) analysis: 3.8 months, 4.8 months, 6.1 months, 9.6 months, 13.7 months, and 31.1 months, respectively; three trials were available for median overall survival (mOS) analysis: 10.2 months, 14.7 months, and not reached, respectively.

**Conclusion:**

CD20×CD3 bispecific antibodies (BsAbs) exhibit efficacy in relapsed or refractory large B-cell lymphoma (LBCL) patients following CAR-T therapy. To validate these findings and determine the optimal sequencing of BsAbs and CAR-T therapy for R/R LBCL patients, prolonged follow-up periods and further prospective clinical trials are warranted.

**Systematic Review Registration:**

https://www.crd.york.ac.uk/prospero/, identifier CRD42024621005.

## Introduction

Major progress has been achieved in the treatment of large B-cell lymphoma (LBCL), including *de novo* diffuse large B-cell lymphoma (DLBCL), primary mediastinal B-cell lymphoma (PMBCL), transformed indolent lymphoma, and high-grade B-cell lymphoma (HGBL). R-CHOP immunochemotherapy (rituximab plus cyclophosphamide, doxorubicin, vincristine, and prednisone) remains the cornerstone of first-line therapeutic regimens (1). However, approximately 10% to 15% of patients treated with R-CHOP exhibit primary refractory disease, and an additional 20% to 25% will experience relapse after an initial response, typically within the first 2 years (2). Autologous hematopoietic stem cell transplantation (ASCT) following high-dose chemotherapy demonstrates clinical efficacy predominantly in chemotherapy-sensitive populations with delayed relapse patterns (3). For patients with primary refractory or early-relapsed hematologic malignancies, chimeric antigen receptor T-cell immunotherapy (CAR-T) shows superior clinical efficacy compared to conventional salvage therapies, particularly in achieving durable remission rates, as evidenced by recent multicenter trials. Follow-up data from the ZUMA-1 study reveal a sustained 4-year overall survival (OS) rate of 44%; however, approximately half of patients achieving a complete response (CR) subsequently relapse (4). The median OS for patients who relapse after CAR-T therapy is 5 to 6 months. In two additional CD19-directed CAR-T therapy trials, TRANSCEND and JULIET, similar overall response rates (ORR) and CR rates were observed.

Bispecific antibodies (BsAbs) represent an innovative immunotherapeutic modality that enhances T-cell activation and tumor cell lysis by simultaneously engaging surface-expressed lymphoma-associated antigens. BsAbs are engineered monoclonal antibodies comprising two distinct domains: one domain binds to CD3 on T cells, while the other targets a tumor-associated antigen. This dual binding induces T-cell-mediated cytotoxicity against the targeted cells (5). Although blinatumomab, a CD19×CD3 BsAb, has been approved for relapsed or refractory (R/R) acute lymphoblastic leukemia, its application in R/R large B-cell lymphoma (LBCL) is limited due to dose-related neurotoxicity (grade ≥3 reported in 22-24% of patients) and efficacy (ORR 37-53%) (5). Additionally, several CD20×CD3 BsAbs have demonstrated promising efficacy in R/R LBCL, including glofitamab, mosunetuzumab, epcoritamab, and odronextamab (5, 6).

Several trials evaluating the role of CD20×CD3 BsAbs in LBCL that relapses or is refractory to CAR-T therapy have been reported. This study aimed to investigate the efficacy of CD20×CD3 BsAbs in patients with R/R LBCL following CAR-T therapy.

## Methods

### Data sources and search strategy

This study has been registered with PROSPERO (CRD42024621005). The data were obtained from reputable medical databases, including but not limited to PubMed, Embase, and Cochrane Library. A comprehensive search strategy was employed using specific medical subject headings (MeSH terms) and keywords relevant to the study objectives.

We systematically searched for research articles in the following databases: PubMed, Web of Science, Scopus, Embase, and Cochrane Library from January 1, 2019, to May 20, 2025. Additionally, we reviewed conference abstracts published by the American Society of Clinical Oncology (ASCO), European Hematology Association (EHA), and American Society of Hematology (ASH).

The search terms were based on MeSH (Medical Subject Headings) as follows: Lymphoma, Large B-Cell, and Antibodies, Bispecific, or ‘Mosunetuzumab’ or ‘Glofitamab’ or ‘Epcoritamab’ or ‘Odronextamab’. For PubMed, the Cochrane highly sensitive search strategy for identifying clinical trial reports was incorporated. Additionally, we manually screened the references of all included trials and reviews to identify further relevant studies.

### Selection criteria

Participants meeting the following inclusion and exclusion criteria were selected for this study. We included full articles and conference abstracts that reported the efficacy of CD20×CD3 bispecific antibodies (BsAbs) in patients with relapsed/refractory (R/R) large B-cell lymphoma (LBCL) after chimeric antigen receptor T-cell (CAR-T) therapy. To prevent data redundancy, cases that appeared in both full papers and conference abstracts were carefully identified and excluded to ensure each case was included only once. Our inclusion criteria were as follows: 1) Studies conducted in adult populations. 2) Studies focusing on patients with R/R LBCL histologically confirmed as large B-cell lymphoma, following CAR-T therapy. 3) Studies evaluating CD20×CD3 BsAbs, including glofitamab, mosunetuzumab, odronextamab, or epcoritamab. 4) Studies with at least 10 patients in the study arm. 5) Studies providing sufficient data, including overall response rate (ORR) or complete response (CR) data. 6) Prospective clinical trials.

### Data extraction and quality assessment

We performed a systematic review and extracted the following study characteristics: publication year, first author’s name, sample size, ClinicalTrials.gov identifier, number of prior treatment lines, rate of refractory to CAR-T therapy, and study design. Furthermore, we collected treatment-related outcomes, including overall response rate (ORR), complete response (CR), overall survival (OS), progression-free survival (PFS), duration of response (DOR), duration of complete response (DoCR), and follow-up duration.

Two reviewers (Zhenyu Zhu and Haobo Ma) independently screened all relevant studies, and any discrepancies were resolved through consensus. The quality of the trials was evaluated using the JBI critical-appraisal checklist for systematic reviews and research syntheses (7).

### Outcome measures

The primary endpoint was the best objective response rate (ORR) and complete response (CR) as assessed by the independent review committee (IRC). Secondary endpoints included investigator-assessed ORR, CR, duration of response (DOR), duration of complete response (DoCR), progression-free survival (PFS), and overall survival (OS). DOR was defined as the time from the initial achievement of ORR until disease progression or death in patients who achieved an ORR. DoCR was defined as the time from the initial achievement of CR until disease progression or death in patients who achieved a CR. PFS was defined as the time from the start of treatment to disease progression or death in all patients, and OS was defined as the time from the first dose of study treatment to death from any cause.

### Statistical analysis

All statistical analyses were performed using STATA software version 16 (StataCorp LP), with the metan and metaprop packages incorporated. A random-effects model was utilized to calculate the pooled estimates of complete response (CR) and overall response rate (ORR), along with their corresponding 95% confidence intervals (CIs) across intervention groups. Between-study heterogeneity was assessed using Higgins’ I² statistic, with the following thresholds for interpretation: ≤25% (low heterogeneity), 26-50% (moderate heterogeneity), and >50% (substantial heterogeneity). Publication bias was assessed using the Egger’s test and funnel plot.

## Results

### Description of trials

**Figure 1:**
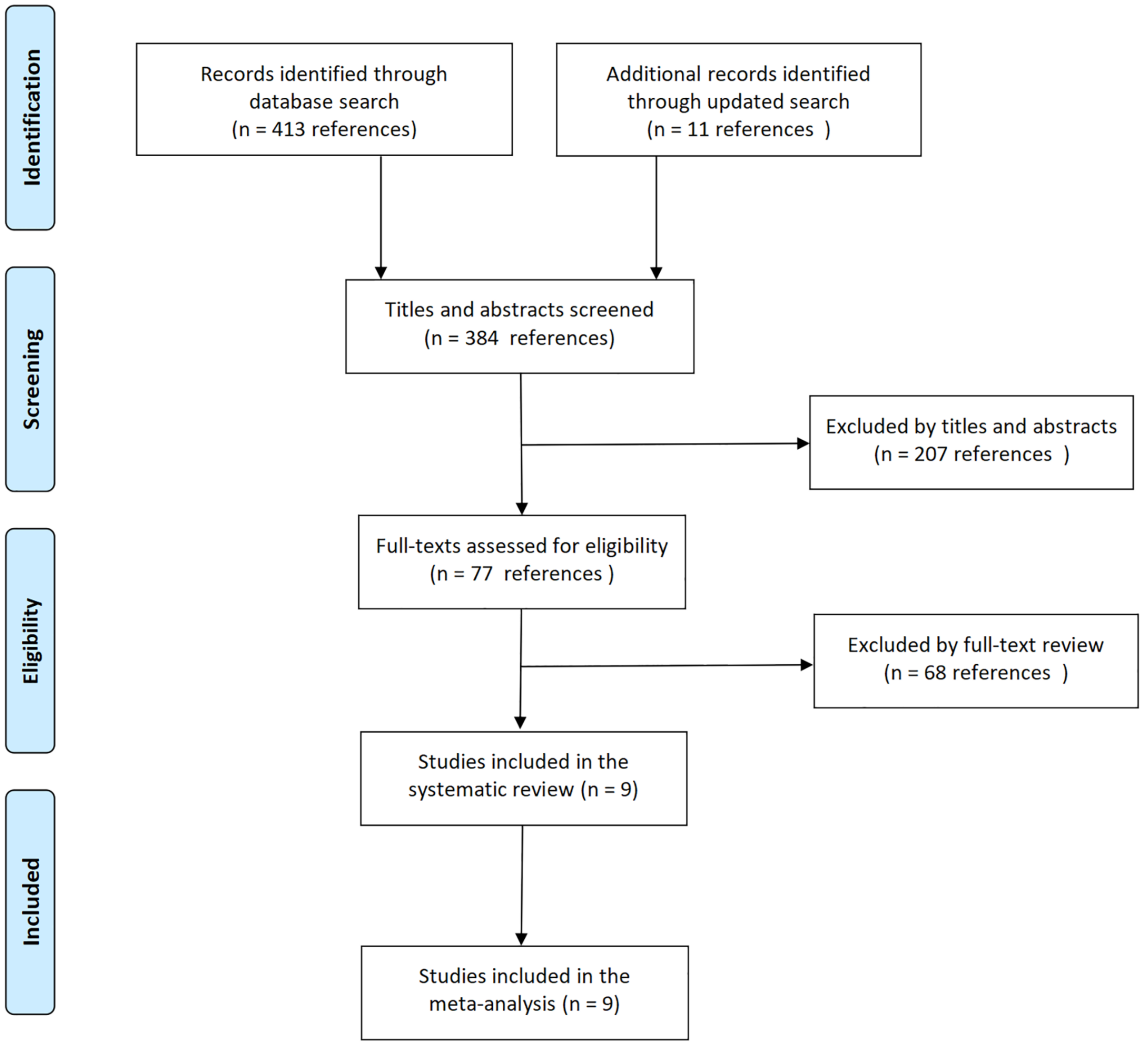
PRISMA flow diagram.

The initial literature search identified 18 relevant records. Following eligibility screening, nine studies met the inclusion criteria, whereas the remaining nine were excluded due to either insufficient post-CAR-T outcome data (n=4) or inadequate sample sizes (<10 patients per cohort, n=5) (**Figure 1**). The systematic review ultimately included seven peer-reviewed articles (8–14) and two conference abstracts (15, 16), collectively comprising nine interventional trials that satisfied the predefined eligibility criteria. The included trials were conducted between 2018 and 2022, with publication timelines extending beyond the study period: results from four trials were reported in 2025, three in 2024, and two in 2023. Two trials addressed mosunetuzumab (8, 9), four trials glofitamab (10, 14–16), two trials epcoritamab (11, 13), and one trial odronextamab (12). Five trials evaluated BsAbs monotherapy (8, 10–12, 15), and four assessed BsAbs combined with immunotherapy or chemotherapy (9, 13, 14, 16). Three trials provided CAR-T-naive data (9, 11, 13).

### Patient characteristics

350 patients were included in our analysis. All patients had previously received CAR-T therapy. CAR-T products included commercial CAR-T (axicabtagene ciloleucel, tisagenlecleucel, lisocabtagene maraleucel), investigational CAR-T (anti-CD19 CAR-T, anti-CD20 CAR-T), and unknown anti-CD19 CAR-T. The proportion of patients who were primarily refractory to prior CAR-T therapy ranged from 66.7% to 88.5%. Intervening therapies administered between CAR-T infusion and BsAbs treatment included no therapy or certain drugs (e.g., bendamustine). The median time from CAR-T infusion to BsAbs initiation varied widely, with the shortest interval recorded being 35 days. Patient ages ranged from 20 to 96 years, and the median follow-up duration was 13 to 42 months. In addition to histologically confirmed large B-cell lymphoma (LBCL), transformed follicular lymphoma, high-grade B-cell lymphoma, and primary mediastinal B-cell lymphoma were also included in the analysis (8–16). **Table 1** summarizes the characteristics of the included patients.

**Table 1:** Characteristics of patients included in trials.

Reference	Clinical identification	Patient number	LOPT	Treatment	Refractory to CAR-T	DOR	DoCR	mPFS	mOS	mFollow up
Chong et al. (8) 2025	NCT02500407	27	3(2-13)	Mosunetuzumab	0.8	1-y 25%	–	6.1m	–	42m
Hutchings et al. (15) 2023	NCT03075696	52	3(2-7)	Glofitamab	0.885	–	22.0m (6.7-NR)	31.1m (22.4-NE)	NE (NE)	32m
Cartron et al. (10) 2025	NCT04703686	46	2(2-6)	Glofitamab	0.667	19.7m (4.0-NR)	NR (19.7-NR)	3.8m (2.4-19.6)	14.7m (8.8-NE)	15.3m
Thieblemont et al. (11) 2024	NCT03625037	61	3(2-11)	Epcoritamab	0.754	9.7m (5.4-NR)	NR	–	–	25.1m
Topp et al. (12) 2025	NCT02290951	60	3(2-9)	Odronextamab	0.717	14.8m (2.8-NR)	NR (3.3-NR)	4.8m (2.6-5.4)	10.2m (4.6-15.8)	16.2m
Hutchings et al. (16) 2023	NCT03533283	27	2(1-7)	Glofitamab + Pola	–	–	–	–	–	13m
Budde et al. (9) 2024	NCT03671018	35	2(1-10)	Mosunetuzumab + Pola	0.743	NR (8.8-NR)	–	9.6m (4.9-NE)	–	23.9m
Brody et al. (13) 2025	NCT04663347	29	2(1-6)	Epcoritamab +Gemox	–	–	–	–	–	13.2m
Abramson et al. (14) 2024	NCT04408638	13	1(1-2)	Glofitamab +Gemox	–	–	–	13.7m	–	20.7m

LOPT, lines of prior treatment; ORR, overall response rate; CR, complete response, DOR, duration of response; DoCR, duration of complete response; mPFS, median progression-free survival; mOS, median overall survival; NR, not reached; NE, not estimated; Pola, polatuzumab vedotin.

### Risk of bias of included trials

**Figure 2:**
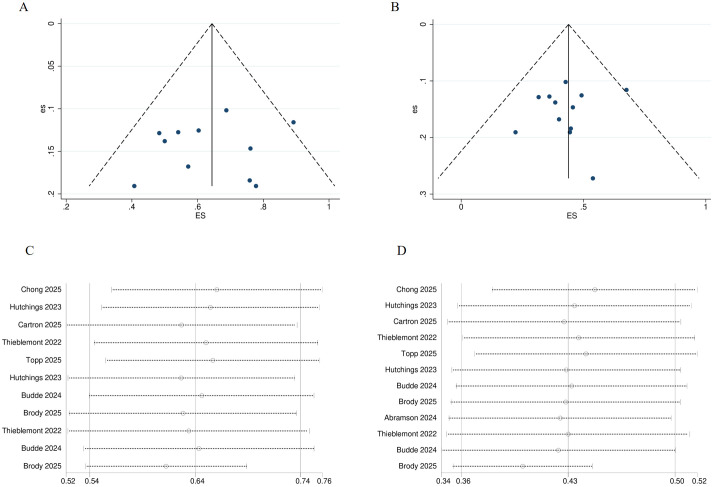
Risk of bias of included trials. **(A)** Funnel plot for ORR. **(B)** Funnel plot for CR. **(C)** Sensitivity analysis of ORR. **(D)** Sensitivity analysis of CR. ORR, overall response rate; CR, complete response.

All trials were single-arm and open-label; however, the primary endpoint was assessed by an independent review committee. Consequently, the risk of attrition bias and selective outcome reporting bias across all trials was minimal. Egger’s test indicated no publication bias for ORR and CR, with *P* = 0.602 and *P* = 0.558, respectively. The funnel plot also confirmed the absence of publication bias for both ORR and CR (See **Figures 2A, B**). Sensitivity analysis of ORR and CR demonstrated that the data were stable (See **Figures 2C, D**).

### Primary outcomes

**Figure 3:**
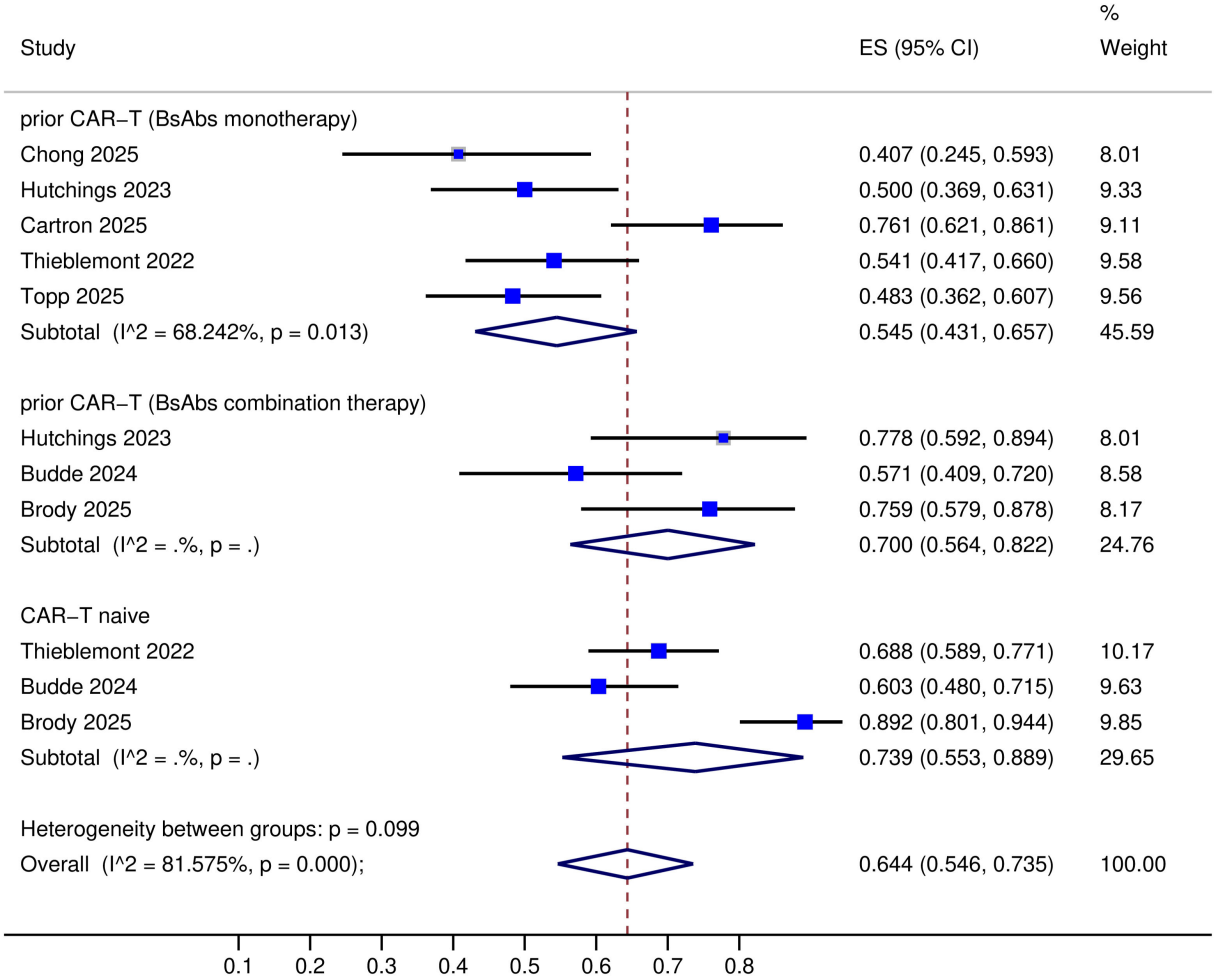
ORR of BsAbs monotherapy or combination therapy prior CAR-T/naive CAR-T.

**Figure 4:**
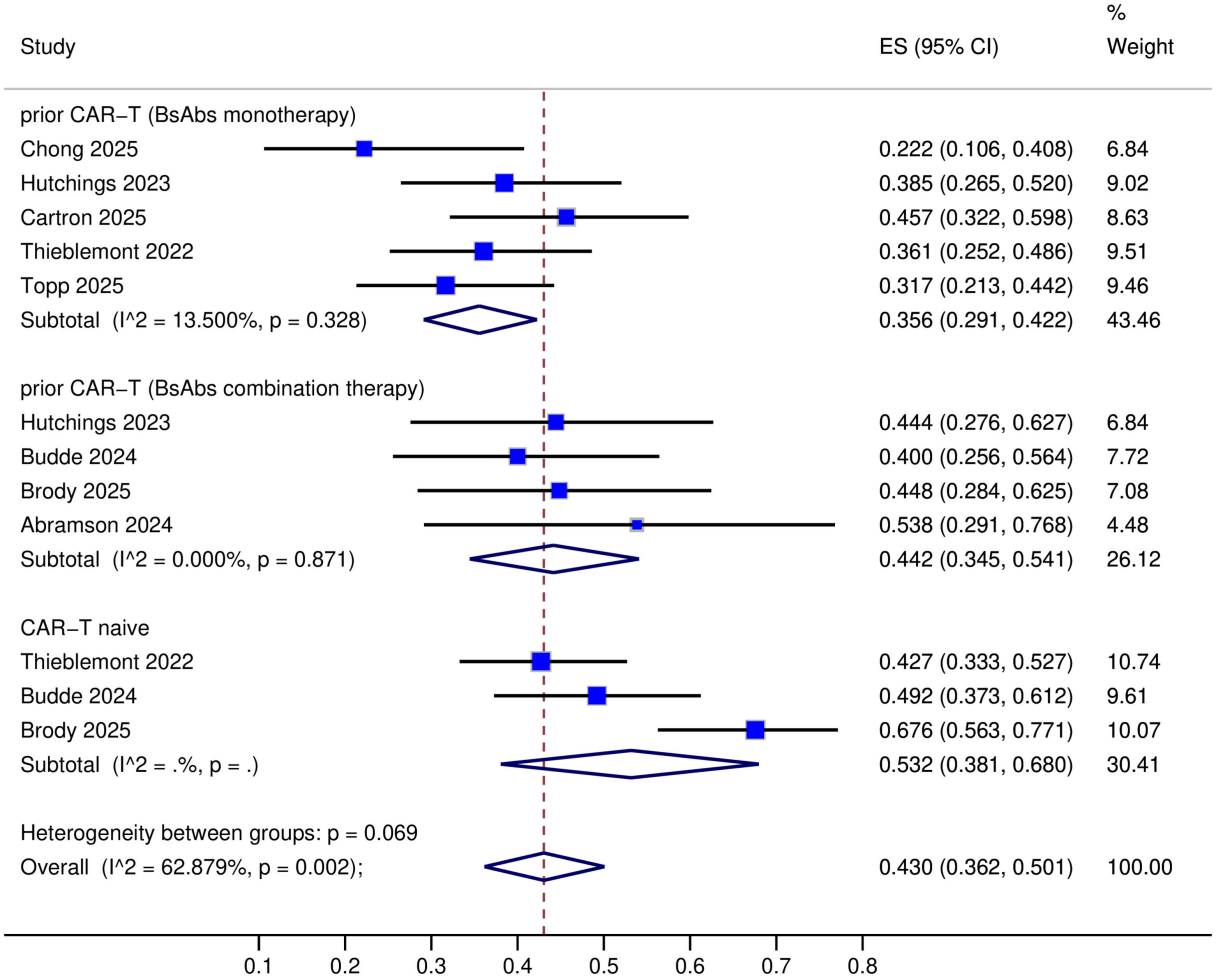
CR of BsAbs monotherapy or combination therapy prior CAR-T/naive CAR-T.

All nine trials provided data on complete response (CR) (8–16), but one trial lacked objective response rate (ORR) data (14). The specific response rates for different BsAbs monotherapies in prior CAR-T therapy R/R LBCL were as follows: Mosunetuzumab: ORR 40% and CR 23%; Glofitamab: ORR 50-76.1% and CR 37-45.7%; Epcoritamab: ORR 54.1% and CR 36%; Odronextamab: ORR 48.3% and CR 31.7% (**Table 2**). Upon pooled analysis, the overall ORR was 54.5% (95% CI: 43.1-65.7%) with significant heterogeneity (*P*=0.013, I²=68.24%), and CR was 35.6% (95% CI: 29.1-42.2%) with low heterogeneity (*P*=0.33, I²=13.5%). The specific response rates for different BsAbs combinations in prior CAR-T therapy R/R LBCL were as follows: Mosunetuzumab + Pola (Polatuzumab vedotin): ORR 57% and CR 40%; Glofitamab + Pola: ORR 77.8% and CR 44.4%; Epcoritamab + Gemox (Gemcitabine plus Oxaliplatin): ORR 76% and CR 45%; Glofitamab + Gemox: CR 53.8% (**Table 2**). Upon pooled analysis, the overall ORR was 70.0% (95% CI: 56.4-82.2%) with no heterogeneity, and CR was 44.2% (95% CI: 34.5-54.1%) with no heterogeneity. (See **Figures 3**, **4**). Three trials were available for the analysis of ORR and CR in CAR-T-naive patients (n=233) (9, 11, 13). The specific response rates for different BsAbs and combinations in CAR-T-naive R/R LBCL were as follows: Epcoritamab: ORR 68.8% and CR 43%; Mosunetuzumab + Pola: ORR 60% and CR 49%; Epcoritamab + Gemox: ORR 89% and CR 68%. Upon pooled analysis, the overall ORR was 73.9% (95% CI: 55.3-88.9%) with no heterogeneity, and CR was 53.2% (95% CI: 38.1-68%) with no heterogeneity. (See **Figures 3**, **4**).

**Table 2:** ORR and CR of patients included in trials.

Reference	Clinical identification	Prior CAR-T	Patient number	LOPT	Treatment	ORR n (%)	CR n (%)
Chong et al. (8) 2025	NCT02500407	Yes	27	3(2-13)	Mosunetuzumab	11(40)	6(23)
Hutchings et al. (15) 2023	NCT03075696	Yes	52	3(2-7)	Glofitamab	26(50)	20(37)
Cartron et al. (10) 2025	NCT04703686	Yes	46	2(2-6)	Glofitamab	35(76.1)	21(45.7)
Thieblemont et al. (11) 2024	NCT03625037	Yes	61	3(2-11)	Epcoritamab	33(54.1)	22(36)
Topp et al. (12) 2025	NCT02290951	Yes	60	3(2-9)	Odronextamab	29(48.3)	19(31.7)
Hutchings et al. (16) 2023	NCT03533283	Yes	27	2(1-7)	Glofitamab +Pola	21(77.8)	12(44.4)
Budde et al. (9) 2024	NCT03671018	Yes	35	2(1-10)	Mosunetuzumab +Pola	20(57)	14(40)
Brody et al. (13) 2025	NCT04663347	Yes	29	2(1-6)	Epcoritamab +Gemox	22(76)	13(45)
Abramson et al. (14) 2024	NCT04408638	Yes	13	1(1-2)	Glofitamab +Gemox	NR*	7(53.8)
Thieblemont et al. (11) 2024	NCT03625037	No	96	3(2-11)	Epcoritamab	66(68.8)	41(43)
Budde et al. (9) 2024	NCT03671018	No	63	2(1-10)	Mosunetuzumab +Pola	38(60)	31(49)
Brody et al. (13) 2025	NCT04663347	No	74	2(1-6)	Epcoritamab +Gemox	66(89)	50(68)

LOPT, lines of prior treatment; ORR, overall response rate; CR, complete response, Pola, polatuzumab vedotin.

Gemox (Gemcitabine plus Oxaliplatin); NR, not reported. * ORR was not explicitly reported in the source but estimated at approximately 75% based on Roche data.

### Secondary outcomes

The median duration of follow-up ranged from 13 to 42 months. Data from five trials were included for DOR analysis, with values of 9.7 months, 14.8 months, 19.7 months, not reached, and 25% at 2 years, respectively (8–12). Three trials were available for DoCR analysis, with one trial reporting 22 months and the others not reached (10–12, 15). Six trials contributed to mPFS analysis, yielding results of 3.8 months, 4.8 months, 6.1 months, 9.6 months, 13.7 months, and 31.1 months, respectively (8–10, 12, 14, 15). Three trials provided data for mOS analysis, with outcomes of 10.2 months, 14.7 months, and not reached, respectively (10, 12, 15) (**Table 1**).

No excess CRS (cytokine release syndrome) or neurotoxicity events were observed in patients receiving either BsAbs monotherapy or combination therapy (9–14). And Cartron et al. assessed health-related QoL (Quality of Life), the mean scores on the functional scales of the EORTC QLQ-C30 (the European Organization for Research and Treatment of Cancer QoL Questionnaire Core 30), including global health status, role functioning and emotional functioning, improved from baseline (day 1 of cycle 1) to day 1 of cycle 3 and were generally sustained in subsequent visits. For the symptom scales, the mean score changes showed improvements in fatigue, pain, dyspnea and insomnia, all of which were also clinically meaningful (10).

## Discussion

CAR-T therapy has transformed the therapeutic landscape for individuals with refractory or relapsed LBCL. Pivotal clinical trials, including TRANSCEND NHL 001, ZUMA-1, and JULIET, demonstrated ORR of 73%, 83%, and 53%, respectively, with CR rates of 53%, 58%, and 39%. Additionally, these trials reported encouraging long-term overall survival rates, ranging from 36% to 42% (4, 17, 18), although approximately half of the patients eventually experienced relapse.

CD20×CD3 BsAbs represent the most promising immunoherapeutics for lymphoma, functioning as novel and accessible T-cell redirecting agents. Monotherapy with BsAbs or in combination with Pola or Gemox achieved overall response rates (ORRs) of 40-76.1% and 57-77.8%, respectively, along with CR rates of 23-45.7% and 40-53.8% in patients with LBCL relapsing after or refractory to prior CAR-T therapy (8–16). Odronextamab monotherapy demonstrated ORRs of 34.9% in a refractory cohort (n=43) and 84.2% in a relapsed cohort (n=17) following prior CAR-T treatment (12). However, prior response to CAR-T therapy was not predictive of subsequent response to mosunetuzumab; among nonresponders (3 of 18, 17%) and responders (3 of 12, 25%) to mosunetuzumab, no significant correlation was observed (8). In different prior CAR-T product groups, odronextamab achieved ORRs of 52.8% (Axicabtagene ciloleucel, n=36), 33.3% (Tisagenlecleucel, n=6), 20% (Lisocabtagene maraleucel, n=10), and 75% (Investigational CD19-directed CAR-T, n=8) (12). Other clinical studies have also included patients who received different types of CAR-T therapy prior to different types of CAR-T before BsAbs treatment (8, 11), however, none specifically reported the efficacy of BsAbs according to the type of prior CAR-T therapy. This is also a limitation of our systematic review. As the number of patients increases in the future, we could further explore the potential impact of CAR-T type on the efficacy of subsequent BsAbs therapy.

In different timeframes for relapse following CAR-T therapy: ≤ 90 days (n=29), 91-≤ 180 days (n=11), 181-≤ 1 year (n=13), >1 year (n=6), Odronextamab achieved ORR of 20.7%, 63.6%, 84.6%, and 83.3%, respectively (12). Chong et al. also investigated the optimal timing and biomarkers for BsAbs after CAR-T therapy. They performed comprehensive analyses using clinical outcome measures and serial blood specimens collected from participants receiving mosunetuzumab therapy following CAR-T therapy (8). No intervening therapy was administered between CAR-T and mosunetuzumab treatment in most patients (20/30), while 10 patients received a single intervening therapy after prior medication washout. Patients who responded to mosunetuzumab treatment demonstrated significantly higher lymphocyte counts and more pronounced increases in CD4+ and CD8+ T-cell populations. Non-responders exhibited a relative reduction in CAR transgene levels (8). Their data indicated that patients with a longer interval between CAR-T infusion and mosunetuzumab initiation were more likely to respond. Additionally, administration of BsAbs 9-12 months following CAR-T therapy was associated with a greater probability of response (8). This study suggests that the timing of lymphocyte recovery following lymphodepleting chemotherapy prior to CAR-T therapy may influence the response to subsequent mosunetuzumab treatment. These findings highlight an association between the outcomes of prior CAR-T cell therapy and the response to subsequent BsAbs therapy, indicating the need for further optimization of the timing for BsAbs administration following CAR-T therapy.

Although both prospective and retrospective studies (19) have demonstrated the efficacy of BsAbs following CAR-T progression, the reverse therapeutic sequence remains underexplored due to exclusion criteria in CAR-T trials that preclude prior BsAbs recipients. It is plausible to hypothesize that prolonged administration of BsAbs therapy may induce T-cell exhaustion, potentially impairing the proliferative capacity and antineoplastic efficacy of CAR-T cells in patient populations previously exposed to BsAbs treatment regimens. The retrospective analysis by Rentsch et al. evaluated CAR-T clonal dynamics during glofitamab therapy in nine CAR-T-experienced patients (20). Pretreatment CAR-T signatures were detected in 55.6% (5/9) of cases, with 33.3% (3/9) demonstrating peripheral CAR-T resurgence peaking at a median of 35 days post-infusion (20). Empirical evidence suggests that the application of BsAbs following CAR-T therapy may represent an efficacious treatment strategy, potentially enhancing the functional capacity of residual CAR-T cells, thus warranting further investigation.

There were two relapse patterns observed following treatment with CD19-directed CAR-T cells: an antigen-positive pattern due to CAR-T cell exhaustion and an antigen-negative pattern. Furthermore, in two additional relapsed cases of mantle cell lymphoma patients treated with CAR-T therapy, a CD19-positive relapse was noted (21). These findings suggest that poor CAR-T persistence and CAR-T cell exhaustion may play a role; meanwhile, an increase in CAR-T cells was observed following administration of glofitamab. Moreover, the optimal sequencing of BsAbs and CAR-T cell therapy remains to be determined.

Several limitations of our analysis warrant consideration. There is a small number of trials on the application of BsAbs following CAR-T therapy, and we included only nine trials, two of which have been published solely as abstracts. These four BsAbs differ in their molecular format, target epitopes, and dosing strategies: mosunetuzumab and epcoritamab are full-length IgG-based 1:1 CD20:CD3 bispecific antibodies, while glofitamab and odronextamab are 2:1 T-cell engaging formats (with distinct binding configurations), which may influence their potency, dosing frequency, and safety profiles. All trials are Phase I/II, except for one Phase III trial. The data regarding DOR, DoCR, and PFS are incomplete.

In the context of precision medicine, the identification and validation of biomarkers have become increasingly critical for early assessment of treatment response, risk stratification, and individualized patient management. In this regard, liquid biopsy, particularly circulating tumor DNA (ctDNA), has emerged as a promising non-invasive tool for monitoring minimal residual disease (MRD), predicting disease relapse, and guiding therapeutic decisions. Recent studies have demonstrated that molecular clustering based on ctDNA mutations can provide superior prognostic stratification in patients with DLBCL, beyond what is achievable by measuring ctDNA levels alone (22, 23). These findings highlight the potential of integrating genomic profiling of liquid biopsy into clinical practice.

In the setting of novel immunotherapies such as CAR-T cell therapy and bispecific antibodies, where deep remission is increasingly achievable, the ability to sensitively and dynamically monitor disease burden is of particular importance. We believe that future prospective studies should focus on validating the role of ctDNA and other liquid biopsy-based biomarkers in this context, to support more precise and timely clinical decision-making.

## Conclusion

In summary, the management of patients with relapsed or refractory LBCL following CAR-T cell therapy remains a critical unmet clinical need in hematologic oncology. Our pooled analysis of nine independent clinical cohorts demonstrated that CD20×CD3 BsAbs exhibit antitumor activity in this challenging patient population, with CR rates ranging from 22% to 46% across studies. Nevertheless, these results are preliminary and subject to substantial methodological limitations inherent in the available evidence base, including heterogeneous patient populations, small cohort sizes, and short median durations of follow-up (ranging from 13 to 42 months). Validation of these preliminary observations and determination of optimal integration strategies between BsAbs and CAR-T cell therapies in relapsed or refractory LBCL will require systematically designed investigations and extended follow-up duration.

## Data Availability

The raw data supporting the conclusions of this article will be made available by the authors, without undue reservation.
